# Data Resource Profile: United Kingdom National Diet and Nutrition Survey Rolling Programme (2008–19)

**DOI:** 10.1093/ije/dyac106

**Published:** 2022-05-28

**Authors:** Michelle C Venables, Caireen Roberts, Sonja Nicholson, Beverley Bates, Kerry S Jones, Robert Ashford, Suzanne Hill, Anila Farooq, Albert Koulman, Nicholas J Wareham, Polly Page

**Affiliations:** Medical Research Council Epidemiology Unit, School of Clinical Medicine, University of Cambridge, Cambridge, UK; Medical Research Council Elsie Widdowson Laboratory, Cambridge, UK; Medical Research Council Epidemiology Unit, School of Clinical Medicine, University of Cambridge, Cambridge, UK; Medical Research Council Elsie Widdowson Laboratory, Cambridge, UK; Medical Research Council Elsie Widdowson Laboratory, Cambridge, UK; NatCen Social Research, London, UK; Medical Research Council Epidemiology Unit, School of Clinical Medicine, University of Cambridge, Cambridge, UK; NatCen Social Research, London, UK; NatCen Social Research, London, UK; Medical Research Council Epidemiology Unit, School of Clinical Medicine, University of Cambridge, Cambridge, UK; Medical Research Council Elsie Widdowson Laboratory, Cambridge, UK; Medical Research Council Epidemiology Unit, School of Clinical Medicine, University of Cambridge, Cambridge, UK; Medical Research Council Elsie Widdowson Laboratory, Cambridge, UK; Medical Research Council Epidemiology Unit, School of Clinical Medicine, University of Cambridge, Cambridge, UK; Medical Research Council Epidemiology Unit, School of Clinical Medicine, University of Cambridge, Cambridge, UK; Medical Research Council Elsie Widdowson Laboratory, Cambridge, UK

**Keywords:** Nutrition survey, dietary assessment, anthropometry, nutritional biomarkers, energy expenditure, population surveillance

Key Features

- The National Diet and Nutrition Survey Rolling Programme (NDNS RP) is a cross-sectional, annual survey designed to collect detailed, quantitative information on food consumption, nutrient intake and nutritional status of the general United Kingdom (UK) population aged ≥1.5 years.
- NDNS RP uses a stratified sampling design to generate a random sample of private UK households each year and a nationally representative core sample of around 1000 participants (500 adults, 500 children) each year. Design provides for additional recruitment at country level.
- Data/samples include: sociodemographic data; dietary assessment; anthropometry; physical activity; energy expenditure; blood and urine samples for nutritional biomarker analysis; National Health Service (NHS) Central Registry and Cancer Registry linkage; contact for further research.
- The NDNS RP dataset (2008–19) comprises: dietary data (7999 adults and 7656 children); blood biomarkers (4181 adults and 2014 children); spot urine (3246 adults and 2318 children); and total energy expenditure (using doubly labelled water) (419 adults and 352 children). Results are published and disseminated via UK Government; survey data accessible via the UK Data Service.
- Stored biological samples are accessible for further health-related research in the public interest through the NDNS Bioresource.
- NDNS RP data underpin monitoring and development of nutrition policy. Data are used for chemical exposure risk assessment and modelling for consumer safety.

## Data resource basics

The purpose of the UK National Diet and Nutrition Survey (NDNS) is to provide detailed quantitative data on food consumption, nutrient intakes and the sources of nutrients for the UK population, and an assessment of population nutritional status through objective biomarker analysis. The UK NDNS reports official statistics for ongoing monitoring of diet, to provide the evidence base for developing and evaluating effectiveness of policy and interventions, and for exposure assessment.

The UK NDNS was set up in 1992 following the first Dietary and Nutritional Survey of British Adults^1^ and was initially conducted as a series of discrete, stand-alone, cross-sectional surveys of different age groups: pre-school children,^2^^,^^3^ older adults,^4^^,^^5^ young people^6^^,^^7^ and adults.^8–10^ From 2008, to increase the ability to track changes over time and enable more rapid response to changing policy needs, the primary data collection moved to a continuous rolling programme (RP) format, sampling across all ages from 1.5 years. This Data Resource Profile focuses on the UK National Diet and Nutrition Survey Rolling Programme (NDNS RP) Years 1–11, 2008–19,^11^ for which the results dataset is publicly available; however, NDNS RP data collection is ongoing and is presently funded to Year 15, 2023. The wider NDNS surveillance programme also includes assessment and monitoring of population salt intakes through adjunct Urinary Sodium Surveys performed periodically for individual UK countries (England, Scotland, Wales and Northern Ireland). For completeness, information about the NDNS Urinary Sodium Surveys (2000–19), past NDNS and other UK nutrition surveys are presented in Supplementary Tables S1 and S2, available as Supplementary data at *IJE* online.

The NDNS RP seeks to collect data from a representative sample of the UK population of around 1000 participants (500 adults, 500 children) each year, with provision for additional representative sampling for country-level analyses in Scotland, Wales and Northern Ireland (‘boost’ sample). The primary aim is the assessment of dietary intake, collected alongside anthropometric, sociodemographic and behaviour variables, following which participants provide blood and urine samples for nutritional status analyses. The NDNS RP has also included doubly labelled water (DLW) sub-studies for measurement of Total Energy Expenditure for assessment of misreporting.

The NDNS RP has employed a dietary assessment method designed to provide full description, quantification and detail of all foods and drinks consumed during the dietary recording period and capable of capturing habitual consumption when conducted over a number of days. Issues of seasonality have been managed through the continuous fieldwork model. Prior to the RP, the age-based surveys used a weighed food diary method (either 4 or 7 days). At the start of the NDNS RP (2008), in order to reduce burden for participants given concerns about falling response rates, an estimated (un-weighed) food diary (collected over 4 consecutive days) was introduced following a dietary methods review and comparison study.^12^^,^^13^ This method was used for 11 years of the NDNS RP (2008–19). From Year 12 (2019), the NDNS RP moved to collect four 24-h recalls (over non-consecutive days) using an automated web-based dietary data collection tool, Intake24 [https://intake24.org/].

**Figure 1:**
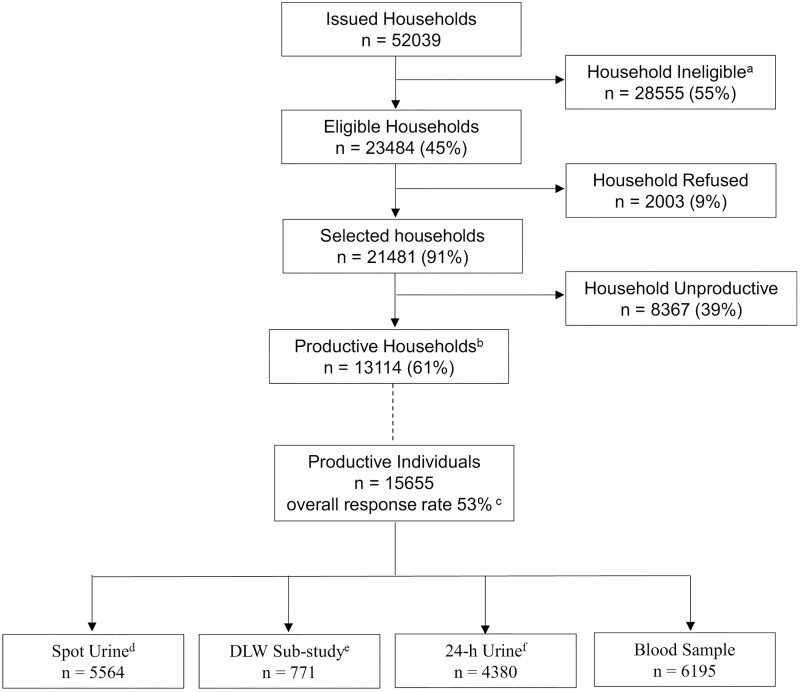
Flow diagram of recruitment numbers and response rates for the UK National Diet and Nutrition Survey Rolling Programme Years 1–11, 2008–2019. DLW, doubly labelled water. ^a^The majority were addresses selected for the child boost sample that were screened out because they did not contain any children in the eligible age range (1.5 to 18 years). The remainder included vacant or derelict properties and institutions. ^b^Those in which one or more participant(s) completed 3 or 4 diary days. ^c^Response is calculation of: the proportion of eligible addresses that are productive multiplied by the proportion of total individuals that are productive. Productive individuals are those that completed 3 or 4 diary days. ^d^Included in Years 6 to 11 for participants aged 4 years and above only. ^e^Included in Years 1, 3, 6 and 7 for participants aged 4 years and above only. ^f^Included in Years 1 to 5 for participants aged 4 years and above only

The overall response rate is based on completion of the dietary assessment element of the survey and ranged from 45.7% to 57.5% for Years 1–11 (Table 1). The published dataset for NDNS RP Years 1–11 includes a total of 7999 adults and 7656 children with dietary information, 4181 adults and 2014 children with blood biomarker data, 4380 participants with 24-h urine collection data (Years 1–5) and 5564 participants with spot urine collection data (Years 6–11) (Figure 1). The survey aimed for ∼10% of the sample to complete the periodic DLW sub-study (conducted every 4–5 years); 771 DLW participants are included in the published dataset.

**Table 1:** Sample size and response in the UK National Diet and Nutrition Survey Rolling Programe Years 1–11 (2008–19)

Core and boost sample combined, unweighted numbers														
Sample type	Response (I)	Response (II)	Response (III)	Survey year										
				Year 1	Year 2	Year 3	Year 4	Year 5	Year 6	Year 7	Year 8	Year 9	Year 10	Year 11
				2008/9	2009/10	2010/11	2011/12	2012/23	2103/14	2104/15	2015/16	2016/17	2017/18	2018/19
				*n*	*n*	*n*	*n*	*n*	*n*	*n*	*n*	*n*	*n*	*n*
Core + country boost	Household response^a^		Issued	5049	5265	5265	5994	3726	4424	4424	4648	4424	4480	4340
			Eligible	2367	2345	2319	2826	1688	1937	1948	2154	1980	2061	1859
			Selected	2128	2132	2133	2583	1490	1748	1721	1860	1905	1987	1794
			Productive	1375	1409	1313	1643	982	1124	1136	1143	1056	1009	924
			Response rate %^b^	56.0	57.5	54.9	55.5	55.7	55.2	55.4	50.2	50.6	45.7	46.7
	Individual response	Adults	Diet^c^	801	812	782	1055	625	663	703	714	647	631	566
			24-h urine^d^	473	513	438	650	431	–	–	–	–	–	–
			Spot urine^d^	–	–	–	–	–	561	588	571	517	523	486
			DLW sub-study^d^	108	–	90	–	–	216	5	–	–	–	–
			Blood	370	450	384	565	372	358	349	355	301	342	335
		Children	Diet^c^	845	857	783	893	572	686	650	656	606	580	528
			24-hour urine^d^	398	410	372	422	273	–	–	–	–	–	–
			Spot urine^d^	–	–	–	–	–	461	402	402	375	347	331
			DLW sub-study^d^	93	–	81	–	–	172	6	–	–	–	–
			Blood	206	231	213	252	158	194	156	173	138	144	149
Country boost	Individual response	Scotland	Diet^c^	306	306	260	501	–	–	–	–	–	–	–
		Wales	Diet^c^	–	167	148	162	158	159	139	199	151	–	–
		Northern Ireland	Diet^c^	209	201	210	202	–	169	161	160	157	149	128

DLW, Doubly Labelled Water. Dash = that element of the study was not included that survey year or no country boost sample for that year. 24-h urine samples were collected in Years 1 to 5, spot urine samples in Years 6 to 11 and the doubly labelled water sub-study was included in Years 1, 3, 6 and 7.

a The classification of eligible/selected/productive households changed over the National Diet and Nutrition Survey Rolling Programme years and may differ slightly to published reports.

b Response is calculation of: the proportion of eligible addresses that are productive multiplied by the proportion of total individuals that are productive. Productive individuals are those that completed 3 or 4 diary days.

c Completed 3 or 4 diary days.

^d^ Ages 4 years and above only.

Survey components are carried out in stages and are voluntary (participants could decline or withdraw at any stage); hence, different numbers of participants have completed individual survey elements. Table 2 provides an age/sex breakdown of all participants for key survey components.

**Table 2:** Profile of participants in the UK National Diet and Nutrition Survey Rolling Programe Years 1–11 (2008–19): all years combined^a^

Core and boost sample combined, unweighted numbers								
Individual response	Age^b^							
	1.5–3 years	4–10 years	11–18 years	Total	19–64 years	65 years	Total	All
				children		and over	adults	participants
	*n*	*n*	*n*	*n*	*n*	*n*	*n*	*n*
Diet								
Male	717	1571	1619	3907	2519	781	3300	7207
Female	658	1440	1651	3749	3617	1082	4699	8448
Total	1375	3011	3270	7656	6136	1863	7999	15 655
24-h urine^c^^,d^								
Male	–	445	509	954	823	237	1060	2014
Female	–	430	491	921	1135	310	1445	2366
Total	–	875	1000	1875	1958	547	2505	4380
Spot urine^d^^,^^e^								
Male	–	642	616	1258	1023	329	1352	2610
Female	–	474	586	1060	1468	426	1894	2954
Total	–	1116	1202	2318	2491	755	3246	5564
DLW^d^^,^^f^								
Male	–	74	104	178	145	59	204	382
Female	–	73	101	174	151	64	215	389
Total	–	147	205	352	296	123	419	771
Blood sample								
Male	76	366	631	1073	1341	393	1734	2807
Female	82	299	560	941	1904	543	2447	3388
Total	158	665	1191	2014	3245	936	4181	6195

a At time of writing, data only published/available for Years 1–11 (2008–18/19).

b The age groups shown are those used for the National Diet and Nutrition Survey Rolling Programme reports.

c 24-h urine samples were included in Years 1 to 5 for participants aged 4 years and above only.

d Some numbers differ from published reports due to the different ways of classifiying ‘obtained samples’.

e Spot urine samples were included in Years 6 to 11 for participants aged 4 years and above only.

f The doubly labelled water sub-study was included in Years 1, 3, 6 and 7 for participants aged 4 years and above only.

NDNS RP conforms to the Declaration of Helsinki and operates under UK National Health Service (NHS) Health Research Authority Research Ethics Committee (REC) Approval (Years 1–5, Oxfordshire REC A, REF 07/H0604/113; Years 6–10 and 11–15, East of England-Cambridgeshire South REC, REF 13/EE/0016).

## Data collected

The NDNS RP is funded by UK Government, currently delivered through the Office for Health Improvements and Disparities, Department of Health and Social Care in England (OHID, DHSC), and the UK Food Standards Agency (FSA). Devolved government departments in Scotland, Wales and Northern Ireland have funded additional NDNS RP recruitment and stand-alone NDNS urinary sodium surveys for their respective countries. The programme is commissioned on a cyclical basis and is currently carried out by a consortium comprising NatCen Social Research (NatCen) and the Medical Research Council (MRC) Epidemiology Unit, University of Cambridge. Interviewer fieldwork in Northern Ireland has been carried out by the Northern Ireland Statistics and Research Agency (NISRA). Survey methods, progress and results publications are overseen by the NDNS Project Board.

### Sampling design

The NDNS RP sampling plan follows a multistage clustered stratified design to generate a new random sample of UK private households each year. The sample has been drawn annually from the ‘small users’ sub-file of the Postcode Address File prepared by the national Post Office. For cost effectiveness, addresses were clustered into small geographical areas, Primary Sampling Units (PSUs), based on postcode sectors randomly selected from across the UK. Sorted first by region, PSUs were then grouped within each region into five equal bands based on Index of Multiple Deprivation score; subsequently, PSUs were sorted by population density. This ensured the sample of addresses was representative of the UK with respect to these three measures. Addresses were then randomly selected from each PSU for the core sample. Additional addresses were selected in Scotland (Years 1–4), Wales (Years 2–9) and Northern Ireland (NI) (Years 1–4 and Years 6–14) to boost respective country sample size for comparisons to be made with the UK as a whole. At each address, the interviewer selected one household at random (if two or more). In order to achieve (as far as possible) equal numbers of adults and children in the sample, at some addresses only children were selected to take part. The interviewer randomly selected up to one adult (aged ≥19 years) and one child (aged 1.5–18 years) from each selected household.

Prior to analysis, weighting factors are applied to all data to account for unequal selection probabilities and non-response so that results are generalisable to the UK population as a whole. The applied weights put the four UK countries into their correct population proportions. In years where recruitment was boosted, the sample design included an adjustment for selecting more addresses in respective individual countries.

### Survey schedule

NDNS RP fieldwork has been organized practically in two stages—interviewer and nurse stages—both undertaken in households of participants (Table 3). The two stages have generally been carried out between 2 to 4 months apart. The interviewer stage comprised three visits/contacts with the household for participant selection and recruitment, administration of the food diary and other protocols (with additional visits for DLW sub-study participants). Participants who completed 3 or 4 diary days were invited to take part in the nurse stage to obtain further physical measurements and a blood sample.

**Table 3:** Survey components used in the UK National Diet and Nutrition Survey Rolling Programme Years 1 to 11 (2008–19)

Survey stage	Component and method	Completed by (age if not completed by all participants)	Survey Years^a^
Interviewer stage	*Computerised Assisted Personal Interview (CAPI):*		
	Household composition, socio	Household representative and	All Years
	economic status, shopping and	main food provider	
	cooking practices		
	Usual eating habits, general health, oral health, dietary supplement use	All participants	All Years
	Sun exposure	All participants	Years 1–10
	Physical activity	Ages 4 to 15 years	Years 6–11
	Consent for NHS Register and Cancer Register data linkage	Age ≥16 years	All Years
	Consent to be contacted for follow-up/further research	All participants (parent/carer on behalf of children)	All Years
	*Self-completion questionnaires:*		
	Smoking and drinking	Age ≥8 years	All Years
	Physical activity	Age ≥16 years	All Years
	Four-day food diary	All participants	All Years
	*Physical measures:*		
	Height and weight	All participants	All Years
	Actigraph (physical activity monitor)	Ages 4 to 15 years^b^	Years 1 to 5
	Spot urine sample	Age ≥4 years	Year 6 onwards
	*Doubly labelled water sub study:*		
	Total energy expenditure	Age ≥4 years	Years 1, 3, 6 and 7
Nurse stage	*Computerised Assisted Personal Interview (CAPI):*		
	Current medication	All participants	All Years
	*Physical measures:*		
	Waist and hip measurements	Age ≥11 years	All Years
	Mid upper arm circumferences	Ages 2 to 15 years	Years 1 to 5
	Infant length	Ages 18 months to 2 years	All Years
	Blood pressure	Age ≥4 years	Years 1 to 10
	Blood samples	All participants	All Years
	24-h urine sample and PABA^c^	Age ≥4 years	Years 1 to 5

^a^ Year 1 2008–09; Year 2 2009–10; Year 3 2010–11; Year 4 2011–12; Year 5 2012–13; Year 6 2013–14; Year 7 2014 15; Year 8 2015–16; Year 9 2016–17; Year 10 2017–18; Year 11 2018–19.

b In Year 1, ActiGraph was only collected for children aged 4 to 10 years.

c Para-aminobenzoic acid.

Italicized text denotes a major component.

### Survey components

Questionnaire information was collected with verbal agreement, and physical measures and biological samples under written consent from participants (aged ≥16 years; parent/carer for younger ages). With consent, a sub-set of biological samples are retained for long-term sample storage. Consent has also been obtained for data linkage to the NHS Central Registry and Cancer Registry and re-contact for further research.

Content and protocols for individual survey components vary with age. Details of questionnaire variables and measurement protocols are regularly reviewed between survey years, and over time some have been modified for improvement, priority, efficiency and/or burden.

Days of food recording are randomly assigned with the aim to evenly represent all days of the week in the NDNS dataset each year. Completed paper food diaries were manually coded using a bespoke diet coding and analysis programme (Diet In, Nutrients Out, DINO).^14^ Coded foods and dietary supplements are linked to food composition data in the NDNS nutrient databank^15^ which draws on information from the Composition of Foods Integrated Dataset (CoFID),^16^ FSA Food Recipes Database^17^ and manufacturers’ data gathered through food labels and web information. Dietary data variables are listed in Supplementary Table S3, available as Supplementary data at *IJE* online.

Height and weight have been measured using portable stadiometer and weighing scales (demispan for ≥65 years, or those for whom there is no reliable height measure) to calculate body mass index (BMI). During the nurse stage, additional physical measures have included seated blood pressure, infant length, waist and hip circumferences and mid upper arm circumferences (Table 3).

For participants aged ≥16 years, following use of a bespoke physical activity (PA) questionnaire during Year 1, the Recent Physical Activity Questionnaire was introduced from Year 2.^18^ For Years 1–10, the PA questionnaires also included sunlight exposure questions.

PA data were initially collected from children aged 4 to 15 years using ActiGraph, a physical device worn at the hip over 7 consecutive days, and subsequently by PA questionnaire based on questions asked in the Health Survey for England [https://digital.nhs.uk/data-and-information/publications/statistical/health-survey-for-england].

Overnight fasted blood samples (non-fasted for ages 1.5–3 years and those unable/unwilling to fast) were collected by venepuncture by a qualified nurse/paediatric phlebotomist and taken by the nurse to a locally recruited laboratory for immediate processing or posted directly to the central NDNS laboratory according to pre-analytical protocol. Following processing and sub-aliquoting, blood samples were stored at −80°C prior to analysis. Urine samples, currently a single spot sample (from Year 6) and previously a 24-h urine sample (Years 1–5), have also been collected for nutritional biomarker analysis from participants aged ≥4 years. All data and biological samples are labelled (link-anonymized, latterly using barcodes) for tracking and traceability.

At intervals, the DLW sub-study was implemented in a sub-sample of participants for measurement of Total Energy Expenditure for assessment of misreporting. Participants were recruited on an age/sex quota basis and provided a pre-dose baseline urine sample and then 10 daily spot urine samples after drinking a body-weight-specific dose of DLW (^2^$H_{2}^{18}$O).

Details of biochemical analyses are provided in Tables 4 and 5. Full details of data and sample collection protocols and analytical procedures are available with published reports and publicly available datasets.

**Table 4:** Blood biomarkers in the UK National Diet and Nutrition Survey Rolling Programe Years 1–11 (2008–19)^a^

Measurement	Sample type	Age (years)	Unit	Assay and comments
Full blood count	Whole blood	≥1.5	Multiple	Beckman Coulter LH700 (Years 1–8); Siemens Advia 2120 (Years 8–11). Performed at Addenbrooke's Hospital
HbA1c^b^	Whole blood	≥1.5	mmol/mol	HPLC (Tosoh Automated Glycohemoglobin Analyser). Years 1–10.
				Performed at Addenbrooke's Hospital
C-reactive protein (CRP)	Serum	≥1.5	mg/L	Siemens Dimension RXL (Years 1–5), Xpand (Years 6–10),
				EXL200 (Year 11) clinical chemistry analysers (manufacturer’s method (extended range)). From Year 11 performed at CBAL
Ferritin	Plasma (Years 1–10), serum Year 11	≥1.5	μg/L	Dade Behring immunonephelometry on Siemens BN ProSpec (Years 1–5), Siemens immunoturbidimetric method on Dimension
				Xpand (Years 6–10), EXL200 (Year 11) clinical chemistry analysers. From Year 11 performed at CBAL
Soluble transferrin receptor (sTfR)	Plasma	≥7	mg/ml	Enzyme immunoassay. Years 1–4
Creatinine	Plasma (Years 1–9), serum Year 10	≥1.5	μmol/L	Siemens Dimension Xpand clinical chemistry analyser (manufacturer’s method). Changed from compensated kinetic Jaffe to enzymatic method during Year 9
Glucose (only if fasted)^b^	Plasma	≥7	mmol/L	Siemens Dimension Xpand clinical chemistry analyser (manufacturer’s method). Years 1–5
Total and HDL cholesterol, triglycerides	Serum	≥1.5	mmol/L	Siemens Dimension RXL (Years 1–5), Xpand (Years 6–10),
				EXL200 (Year 11) clinical chemistry analysers (manufacturer’s methods). From Year 11 performed at CBAL
LDL cholesterol	Serum	≥1.5	mmol/L	Calculated (Friedewald equation)
Thiamin (vitamin B_1_)	Red blood cell haemolysates	≥1.5	n/a	ETKAC
Riboflavin (vitamin B_2_)	Red blood cell haemolysates	≥1.5	n/a	EGRAC
Vitamin B_6_ (pyridoxal-5-phosphate and pyridoxic acid)	Plasma	≥1.5	nmol/L	HPLC with fluorimetric detection
Vitamin B_12_	Serum	≥1.5	ng/L	Siemens Advia Centaur (manufacturer’s competitive protein binding method). Performed at Addenbrooke's Hospital
Holo-transcobalamin	Serum	≥1.5	pmol/L	ELISA (Axis Shield Diagnostics, Dundee). Years 6–11
Homocysteine	Plasma	≥7	μmol/L	Immunoturbidimetric assay on Siemens BN ProSpec (manufacturer’s method). Years 1–5
Folate	Serum	≥1.5	nmol/L	LC-MS/MS (During Years 1–6 performed at CDC)
Whole blood folate	Whole blood	≥1.5	nmol/L	Microbiological method, performed at CDC. Red cell folate is calculated from whole blood folate, serum folate and haematocrit.
Vitamin C	Plasma	≥1.5	μmol/L	Fluorescent enzymatic assay
Retinol (vitamin A), retinyl palmitate, α-tocopherol, γ-tocopherol (vitamin E), α- and β-cryptoxanthin, lycopene, lutein and zeaxanthin (combined), α- and β –carotene	Plasma	≥1.5	μmol/L	HPLC with photodiode array detection. Retinyl palmitate measured in Years 1–4 only
Vitamin D (25-hydroxyvitamin D)	Serum	≥1.5	nmol/L	CLIA (DiaSorin Liaison (Years 1–6)). LC-MS/MS (Years 7–11)
Selenium and zinc	Plasma (Years 1–10), serum Year 11	≥7	μmol/L	Inductively coupled plasma mass spectrometry (ICP-MS). From
				Year 11, performed at University Hospital Southampton. Measured in all ages from Year 11
Repository samples	Plasma/serum	≥1.5	n/a	

CBAL, Core Biochemical Assay Laboratory, Addenbrooke's Hospital; CDC, Centers for Disease Control; CLIA, Chemiluminescence immunoassay; EGRAC, erythrocyte gluthathione reductase activation coefficient; ELISA, enzyme-linked immunosorbent assay; ETKAC, erythrocyte transketolase activation coefficient; HbA1c, glycated haemoglobin; HDL, high density lipoprotein; HPLC, high performance liquid chromatography; LC-MS/MS, liquid chromatography tandem mass spectrometry; LDL, low density lipoprotein [Friedewald equation: LDL = Total cholesterol – HDL cholesterol – (triglycerides/2.2)].

a Analyses were peformed at Medical Research Council Elsie Widdowson Laboratory (Years 1–10) or Nutritional Biomarker Laboratory, MRC Epidemiology Unit (Year 11 onwards) unless otherwise stated.

b Analysis of these analytes was funded separately by Diabetes UK.

**Table 5:** Urine biomarkers in the UK National Diet and Nutrition Survey Rolling Programe Years 1–11 (2008–19)^a^

Measurement	Age (years)	Unit	Assay
24-h urine:			Years 1–5 only
Sodium, potassium	≥4	mmol/L	Ion-specific electrode (Integrated Multisensor Technology) on
			Siemens Dimension Xpand (manufacturer's method)
Creatinine, urea	≥4	mmol/L	Siemens Dimension Xpand clinical chemistry analyser (manufacturer’s method). Changed from compensated kinetic Jaffe to enzymatic method during Year 9
Para-amino benzoic acid	≥4	mg/L	High-performance liquid chromatography with UV detection
Nitrogen	≥4	g/L	FP-428 LECO Nitrogen Determinator, performed at Institute of Grassland and Environmental Research (IGER), now the Institute for Biological, Environmental and Rural Sciences (IBERS),
			University of Aberystwyth, UK.
Repository samples	≥4		
Spot urine:			Years 6–11 only
Iodine	≥4	μmol/L	Inductively coupled plasma mass spectrometry, performed at
			University Hospital Southampton
Repository samples	≥4		
Doubly labelled water:^b^			Years 1, 3, 6 and 7 only
TEE	≥4	kcal/day	Isotope ratio mass spectrometry: Platinum equilibration and CO_2_ equilibration

TEE, total energy expenditure; UV, ultra-violet.

a Analyses were peformed at Medical Research Council Elsie Widdowson Laboratory (Years 1–10) or Nutritional Biomarker Laboratory, MRC Epidemiology Unit (Year 11 onwards) unless otherwise stated.

^b^ CO_2_ production rate was calculated using the multipoint method of Schoeller *et al.*,^19^ a food quotient from the diet tool was calculated using Jequier *et al*.^20^ and converted to total energy expenditure using the energy equivalent of CO_2_ from Elia and Livesey.^21^

Participants received monetary gift vouchers following completion of individual survey components and were offered written feedback on height, weight and BMI (≥16 years), dietary intake, blood pressure and, with written consent for themselves and/or their General Practitioner, results of clinically relevant biomarkers.

Survey personnel (including fieldwork, data analysts, biochemical analysts) are recruited with specialist expertise and receive training including demonstration and practice sessions, quality control and competency assessments (e.g. anthropometry, dietary assessment, sample collection, bioanalysis). Fieldworker training has been undertaken at the beginning of each new fieldwork year, with refresher and additional briefing training at intervals throughout the year. Data checks for reconciliation, quality control and assurance are carried out according to standard operating procedures. Laboratory assays include gold-standard methodologies, use of internal quality controls to monitor performance over time and, where available, participation in external quality assessment schemes to monitor performance and/or accuracy (dependent on the scheme).

### Data collection from Year 12

NDNS data collection is ongoing. From Year 12 (October 2019), in response to methodological and scientific advances and challenges including the drive for cost effectiveness,^22^^,^^23^ dietary assessment changed from paper-based food diary to online 24-h recalls using Intake24. In parallel, PA questionnaires also moved online and, for efficiency, participant selection within a household increased. An evaluation of methodological changes is in progress.^24^

In response to the global COVID-19 pandemic, fieldwork was initially suspended in March 2020 and resumed in October 2020 with an adapted remote fieldwork model enabling continued data collection for questionnaire-based variables and self-reported physical measures and a DLW sub-study; collection of blood and spot urine samples resumed in October 2021. The next NDNS RP data release is expected in 2023.

## Data resource use

NDNS RP results are regularly published via Government in paired or combined data years for reasons of sample size. Publications include summary statistics for the UK as a whole^25–31^ and the devolved countries, Scotland,^32^ Northern Ireland^33^^,^^34^ and Wales,^35^^,^^36^ separately. The next NDNS RP results publication is expected in 2023 to follow completion of Years 12–14 (2019–22).

NDNS RP reports are published as Official Statistics and are regularly used by government, researchers, health professionals and industry to provide evidence on diet, nutrient intake and nutritional status in UK population age/sex groups.^37^ They underpin policy development and monitoring of diet and nutrition objectives, such as UK Government’s long-term initiatives to reduce childhood obesity,^38^^,^^39^ the calorie reduction programme,^40^ introduction of the 2018 UK soft drinks industry levy^41^ and the recent decision to introduce mandatory folic acid fortification of flour [https://www.gov.uk/government/consultations/adding-folic-acid-to-flour/outcome/proposal-to-add-folic-acid-to-flour-consultation-response]. Over the past 20 years, NDNS data have informed reports and position statements of the UK Scientific Advisory Committee on Nutrition,^42–46^ feeding into such policy development. Data are used by the FSA for risk assessments and consideration of food safety implications, including dietary contaminants and toxicology exposure modelling and assessing implications of novel foods entering the UK food chain. NDNS data contribute to global health through international datasets (e.g. the European Food Safety Authority, World Health Organization, Food and Agriculture Organization of the United Nations) and initiatives such as the Vitamin D Standardisation Programme and identification of high prevalence of vitamin D deficiency throughout the European population.^47^ NDNS RP data have also informed development of guidelines for interpretation of micronutrient biomarkers and risk factors for anaemia.^48^^,^^49^ The NDNS RP DLW data provide a unique nationally representative reference dataset for the UK population and have recently been used to describe and estimate components of energy expenditure.^50^

## Strengths and weaknesses

The NDNS RP provides a unique source of nationally representative, highly detailed, quantitative information on food consumption, providing reliable estimates of usual dietary intakes and distributions, with blood and urine samples providing objective evidence of nutritional status for the UK population. The continuous nature of the Rolling Programme provides a powerful opportunity for time trend analyses of nutritional health and wellbeing across the age spectrum.^28^ The collection of detailed anthropometric, sociodemographic, economic and other contextual and behavioural information enables analysis by population sub-groups when data years are combined. NDNS RP reports include detailed analyses by age and sex, and previous reports have included comparison of intakes and status by income and other indicators of deprivation.^25^^,^^28^^,^^32–36^ The availability of this comprehensive dataset and access to biological samples through the NDNS Bioresource offers potential for further research in the public interest and scope for data linkage at the individual level.

As with all dietary surveys and studies, assessment of usual diet relies on self-report instruments that can be prone to measurement error and bias. The use of a comprehensive and detailed dietary assessment instrument conducted over 3 to 4 days accounts for day-to-day variation and provides potential to adjust for dietary data for usual intakes. Along with regular DLW assessments carried out within the survey sample for periodic quantification of misreporting of energy, this enables comprehensive measurement of diet and understanding of data limitations. The rigour of NDNS survey methods and quality of resulting data are underpinned by gold-standard nutritional biomarker methods and investment in international initiatives for data harmonization. Furthermore, the complex sample design, intensive fieldwork model and weighting strategy all help to ensure that data are representative of the UK population.

Similar to the picture seen in other national surveys,^51^ NDNS RP response rates have declined in recent years. An internal review in 2018 found no substantive evidence linking the decrease in response rates to a possible increase in bias of NDNS RP estimates.

Long-running surveillance programmes depend on standardized measurement with continuity over time to provide comparability of data between assessments. However, it is also vital that survey methods remain fit for purpose to ensure relevance and efficiency. Where methods are subject to change within the NDNS RP, implications are assessed to identify aspects of data discontinuity with previous years of the NDNS RP, including through evaluation and cross-validation studies where feasible to facilitate data harmonization.

## Data resource access

NDNS RP reports are openly available and published regularly via [gov.uk]. Anonymized individual-level data are available to registered users through the UK Data Service (UKDS). NDNS RP biological samples are accessible via the NDNS Bioresource. For enquiries contact Polly Page [polly.page@mrc-epid.cam.ac.uk].

## Ethics approval

The NDNS RP conforms to the Declaration of Helsinki and operates under UK National Health Service (NHS) Health Research Authority Research Ethics Committee (REC) Approval (Years 1–5, Oxfordshire REC A, REF 07/H0604/113; Years 6–10 and 11–15, East of England-Cambridgeshire South REC, REF 13/EE/0016).

## Supplementary Material

dyac106_Supplementary_Data

## Data Availability

See Data Resource Access, above.
